# Natural history and literature review of primary intracranial CIC-rearranged sarcomas: case report

**DOI:** 10.3389/fonc.2026.1958689

**Published:** 2026-10-12

**Authors:** Arissa Khan, Maryam Khan, Abigail Bowser, Sahara Cathcart, Nicole Shonka, Sean Kelly, Daniel Surdell, Breann Bowar, Danielle Cunningham, Qateeb Khan

**Affiliations:** 1 Case Western Reserve University, Cleveland, OH, United States; 2 Medical College of Wisconsin, Milwaukee, WI, United States; 3 Department of Radiation Oncology, University of Nebraska, Omaha, NE, United States; 4 Department of Pathology, University of Nebraska, Omaha, NE, United States; 5 Department of Medical Oncology, University of Nebraska, Omaha, NE, United States; 6 Department of Radiology, University of Nebraska, Omaha, NE, United States; 7 Department of Neurosurgery, University of Nebraska, Omaha, NE, United States; 8 Department of Radiation Oncology, University of Kansas, Kansas City, KS, United States

**Keywords:** brain cancer, intracranial sarcoma, neurooncology, radiothearpy, sarcoma

## Abstract

**Background:**

Intracranial sarcomas are particularly rare primary central nervous system malignancies. Here we report the complete oncologic course of a patient diagnosed with a primary intracranial CIC-rearranged sarcoma with subsequent intracranial spread.

**Case presentation:**

A 36-year-old female patient with a prior history of melanoma presented to the emergency department with sudden-onset headaches and paresthesia. After workup demonstrated an intracranial lesion, she underwent gross total resection, with pathology showing primary intracranial sarcoma. She completed adjuvant radiation with an excellent local response. Unfortunately, she developed distant intracranial parenchymal lesions, managed by further resection, radiotherapy, and systemic therapy. One year after initial diagnosis, she presented with profoundly altered mental status with both local and distant intracranial failure, and she passed away a few days later.

**Conclusion:**

CIC-rearranged sarcomas are very rare malignancies and should be managed by a multidisciplinary team. While aggressive local failures are reported in these malignancies, here we report an anomalous pattern of rapid distant intracranial spread.

## Introduction

CIC-rearranged sarcomas (CRS) are particularly rare mesenchymal tumors characterized by rearrangements involving the CIC (Capicua transcriptional repressor) gene. Historically classified as Ewing-like sarcomas, given some morphologic similarities, they are now a separate entity altogether with unique features and a poorer prognosis (1). Most commonly arising in the soft tissues of the trunk and extremities, they carry a high risk of local recurrence despite aggressive multimodal therapy (2). Primary intracranial CIC-rearranged sarcomas are exceedingly rare, with only a handful of cases reported in the literature (3). Surgical resection remains the cornerstone of management, often followed by adjuvant radiotherapy and occasionally systemic therapy. There are no standardized treatment guidelines for the management of this malignancy. Here, we present a case of a primary intracranial CIC-rearranged sarcoma, with an emphasis on the clinical presentation and the patient’s subsequent oncologic course. Written informed consent for publication was obtained.

## Case description

A 36-year-old female patient with a past medical history significant for T1aN0M0 melanoma of the left thigh status post wide local excision alone with negative margins 4 years earlier, presented to the emergency department (ED) with an 8-day history of new-onset headaches, nausea, brain fog, and left arm paresthesia. CT of the head showed a right temporal lobe mass; subsequent MRI demonstrated a 1.7 cm enhancing intra-axial mass with surrounding vasogenic edema and associated mass effect (**Figure 1**). Given the concern for metastatic disease, systemic imaging using both CT of the chest, abdomen, and pelvis (CAP) and PET CT showed no evidence of malignancy apart from the intracranial lesion. She therefore underwent craniotomy and tumor resection. Due to the concern for intracranial metastatic disease from her prior melanoma, Gliadel (carmustine) wafers were placed at the time of surgery. She tolerated the surgery well and was discharged home.

**Figure 1:**
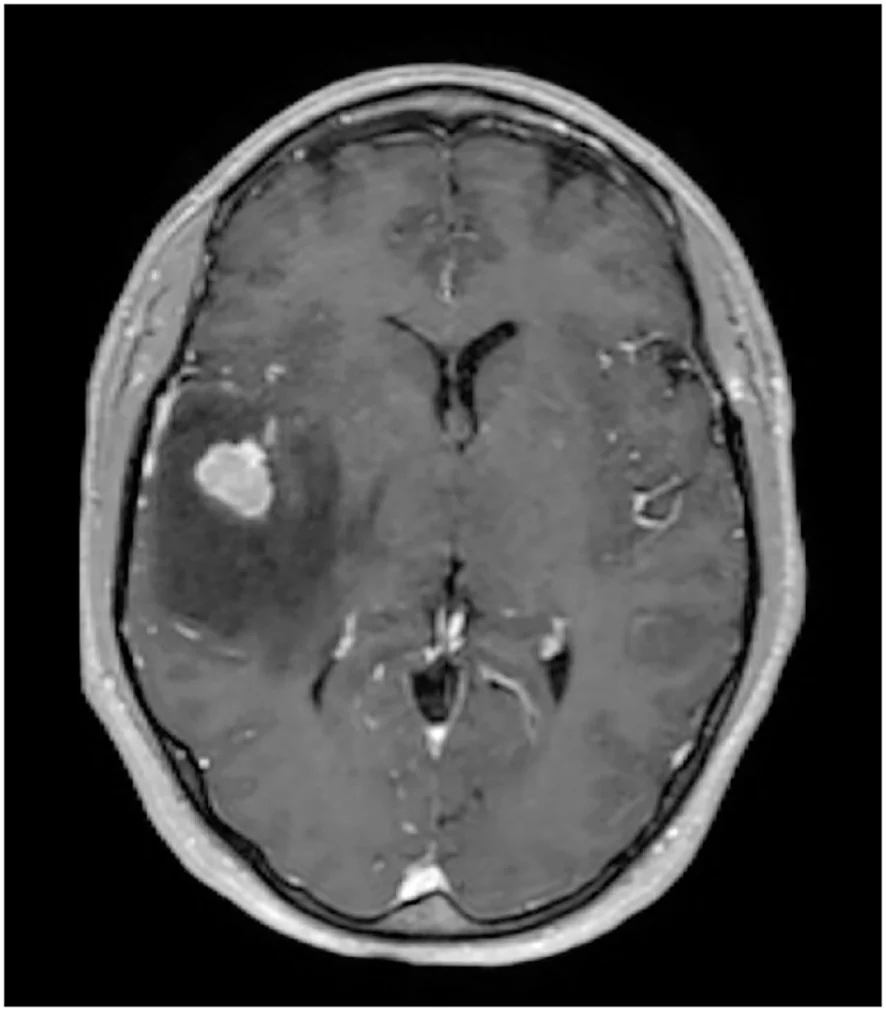
Axial post-contrast T1-weighted MRI demonstrates a 1.7 cm homogeneously enhancing mass with prominent vasogenic edema and regional mass effect.

Her case was discussed at a multidisciplinary neuro-oncology tumor board. Pathology demonstrated that the tumor exhibited poorly differentiated high-grade morphology with a solid growth pattern. The tumor was positive for CD99, TLE1, vimentin, SMA (patchy), and WT1 (patchy), with overexpression of p53 and focal/patchy weak Olig2 expression (**Figure 2**). Markers for melanoma, carcinoma, hematopoietic neoplasia, and various other markers were negative. The tumor morphology and immunoprofile were most suggestive of a primary high-grade central nervous system (CNS) sarcoma. Tumor methylation profiling (National Institutes of Health, Infinium MethylationEPIC kit, Illumina) demonstrated a high-confidence match to the methylation class “CIC-rearranged sarcoma,” and next-generation sequencing (UCSF500 Cancer Gene Panel with reflex RNA whole transcriptomic sequencing) revealed an ATXN1::DUX4 fusion. Consensus was for adjuvant radiotherapy, which was delivered via volumetric modulated arc therapy (VMAT) at 60 Gy in 30 fractions. She completed this course well and underwent subsequent surveillance imaging that showed no evidence of residual or recurrent tumor.

**Figure 2:**
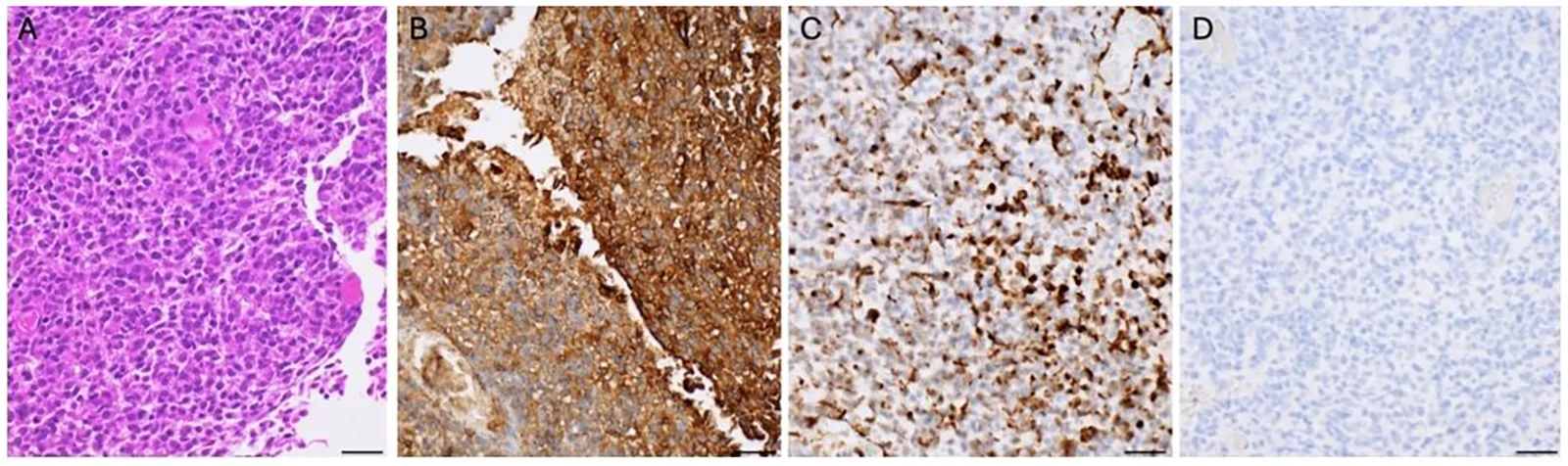
Histologic review of the right temporal tumor demonstrates a poorly differentiated malignant neoplasm with frequent mitotic images [**(A)**, H&E, 400x; arrows highlight mitotic images]. Select immunostains show that the tumor is diffusely positive for CD99 [**(B)**, 400x], shows patchy WT1 expression [**(C)**, 400x], and is negative for NKX2.2 [**(D)**, 400x]. Scale bars = 20 µm.

Unfortunately, 4 months after she completed radiotherapy, she again presented to the ED with a 10-day history of frontal headaches. A brain MRI showed a new 4.4 cm heterogeneously enhancing hemorrhagic right frontal lobe mass with significant vasogenic edema, mass effect, and leftward midline shift (**Figure 3**). The previously resected and irradiated cavity was entirely stable with no evidence of local recurrence. CT CAP demonstrated no evidence of systemic disease and cerebrospinal fluid (CSF) cytology was negative. She subsequently underwent another gross total resection. The tumor demonstrated morphology and immunophenotype similar to those of the initial resection specimen, consistent with recurrent disease, spatially separate from the initial site of involvement.

**Figure 3:**
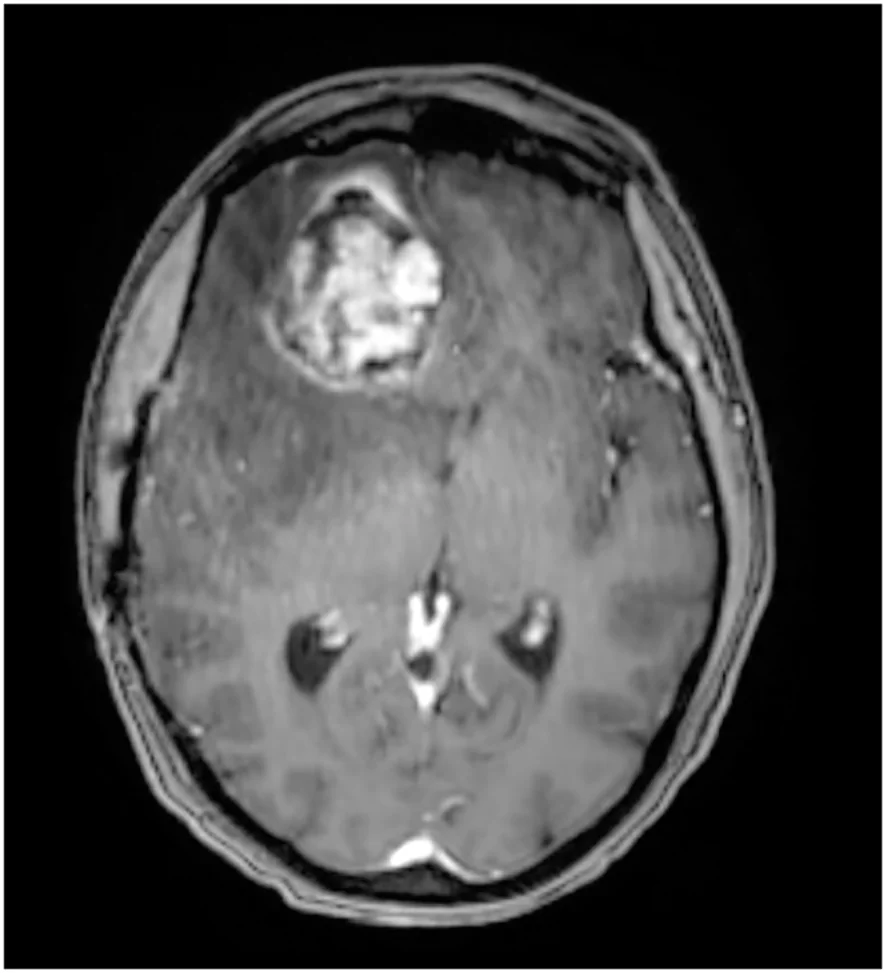
Axial post-contrast T1-weighted MRI demonstrates a new 4.4 cm heterogeneously enhancing right frontal lobe mass clearly separate from the original treatment site with significant vasogenic edema and mass effect, including sulcal effacement, ventricular distortion, and leftward midline shift.

Given her previous excellent response, her case was again discussed at a multidisciplinary neuro-oncology tumor board with consensus for radiotherapy. Radiotherapy options were presented for this isolated area of disease, including repeating a conventional course of photon radiotherapy, a hypofractionated course of photon radiotherapy, and referral for protons to minimize risk of radionecrosis. She elected to undergo proton radiotherapy. Unfortunately, during the simulation scans for proton radiotherapy, she was found to have local recurrence at her second operative site. After further multidisciplinary discussion, she began hypofractionated proton radiotherapy with a planned dose of 40.05 Gy in 15 fractions. However, after 2 fractions, she began to decline neurologically with altered mental status. Her family elected to return home; she continued steroids and completed a course of adjuvant photon radiotherapy to 34 Gy in 10 fractions. She tolerated radiotherapy well and had significant cognitive improvement.

Brain MRI performed 1 month after radiotherapy showed an excellent response of the irradiated lesion with decreased size of the mass, midline shift, and near-complete resolution of solid enhancing intralesional components. There was a small residual nodular component along the anteromedial margin of the mass. The absence of elevated perfusion within the treated lesion supported treatment response rather than recurrent tumor. Unfortunately, a new homogeneously enhancing solid mass in the left middle cranial fossa measuring 2.4 x 2.0 x 1.8 cm had developed and showed elevated perfusion parameters, concerning for a third disparate site of disease (**Figure 4**). Given this pattern of intracranial spread, multidisciplinary consensus was for radiotherapy followed by systemic therapy. Whole-brain radiotherapy and stereotactic options were discussed; she elected to undergo stereotactic treatment of the isolated area of new disease. Given the lesion size and proximity to the optic apparatus, fractionated stereotactic radiotherapy (fSRS) was delivered at 30 Gy in 5 fractions. Inpatient ifosfamide and pazopanib were offered adjuvantly. She elected to start with pazopanib to maximize her time as an outpatient; pazopanib was started 1 week after fSRS. Brain MRI 3 weeks later showed decreased size of the third irradiated lesion and no new sites of disease.

**Figure 4:**
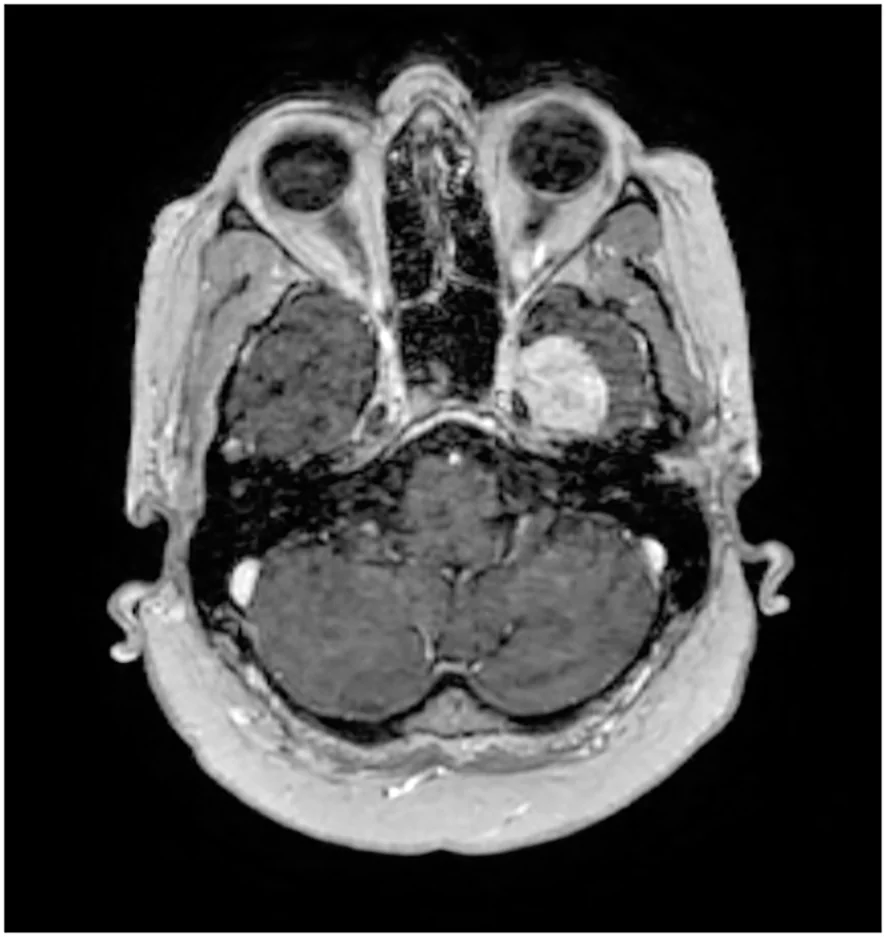
Axial post-contrast T1-weighted MRI demonstrates a new, distant, homogeneously enhancing mass in the left middle cranial fossa measuring 2.4 x 2.0 x 1.8 cm.

Around 6 weeks after initiation of systemic therapy, she presented to the emergency department with altered mental status and failure to thrive. Brain MRI showed interval progression of intracranial spread: both local and distant. The second irradiated site, the right anterior frontal lobe mass, was now crossing the midline, and there were at least 6 new enhancing masses. The first and third irradiated sites were stable with no local progression. Therapeutic options were discussed, including whole-brain radiotherapy and stereotactic radiotherapy. Given her significantly altered mental status and failure to thrive, the patient and her family elected to pursue comfort measures with no further oncologic care. She succumbed to her disease a few days afterwards.

## Discussion

CIC-rearranged sarcomas (CRS) are rare, highly aggressive mesenchymal tumors, accounting for fewer than 1% of all sarcomas. Previously classified within the Ewing family of sarcomas, they are now recognized as a distinct entity under the 2020 WHO classification of bone and soft tissue tumors (4). They typically affect younger adults, with a range of median ages between 25 to 35 years; these tumors most commonly arise in the soft tissues of the extremities and trunk (2). The prognosis of CRS is poor, as seen in our case. In a large study of over 100 cases by Antonescu et al., the 5-year overall survival (OS) was only 43%, significantly lower than the 77% observed in age- and stage-matched Ewing sarcoma controls (p = 0.002) (5). Similarly, in another global study of over 150 cases, the GRACefUl collaborative group reported a 3-year OS of only 39.6% for CIC-rearranged sarcomas (6). For primary intracranial CRS, no large cohort studies exist, and reported survival times have been limited in the few published cases (7, 13).

On MR imaging, CRS typically appear as large masses with central necrosis, heterogeneous contrast enhancement, surrounding mass effect, and vasogenic edema. This imaging appearance of primary intracranial CIC-rearranged sarcoma is nonspecific. In this case, the lesions manifested as homogeneously enhancing intra-axial masses with prominent vasogenic edema, mass effect, and hemorrhagic change. These imaging findings overlap considerably with hemorrhagic metastases, gliomas, and non-neoplastic processes, underscoring the importance of histopathologic and molecular characterization for definitive diagnosis. Advanced MRI techniques, including perfusion imaging, were useful during surveillance, as the absence of elevated relative cerebral blood volume within treated lesions favored post-treatment change, whereas elevated perfusion in newly developed lesions supported recurrent tumor. Grossly, these tumors tend to be large, circumscribed, white/tan masses, with frequent necrosis and hemorrhage. Histologically, CRS is characterized by sheets of small round cells with coarse chromatin, conspicuous nucleoli, and variable amounts of clear to pale eosinophilic cytoplasm often with clearing (8). Stromal myxoid change may be seen in some cases. More commonly, there is a high mitotic rate and geographic necrosis with perivascular preservation. Immunohistochemically, these tumors may demonstrate patchy membranous CD99 expression—in contrast to the strong diffuse pattern seen in Ewing sarcoma—along with nuclear WT1 positivity (>90% of cases), diffuse ETV4 expression, and DUX4 positivity. NKX2.2, a fairly reliable marker for Ewing sarcoma, tends to be negative in CRS (9). In the current era, molecular confirmation through fluorescence *in situ* hybridization (FISH), RNA sequencing, or next-generation sequencing is essential for definitive diagnosis, particularly in unusual anatomic locations such as the central nervous system, as in our patient. Of specific relevance to our patient, ATXN1::DUX4 fusions have recently been identified as molecular events in this sarcoma subtype. Within the past 3 years, both an adult case of CNS sarcoma with ATXN1::DUX4 fusion and a soft tissue case of this entity were reported (10, 11). In the former case, reported by Satomi et al., a 40-year-old male patient presented with a right frontal lobe mass and passed away around 1 year after initial presentation due to aggressive local failure. In our report, we demonstrate that with aggressive multimodal oncologic approaches, control may be achieved at the primary site, but intracranial spread may occur.

As with most sarcomas, the cornerstone of oncologic treatment is surgery. In locations other than the central nervous system, there are occasional long-term survivors among patients who undergo complete surgical resection with negative margins. In the Canadian Sarcoma Research and Clinical Collaboration (CanSaRCC) study, no patients treated without surgery survived long-term, and only surgical resection was associated with improved overall survival (4). Similarly, in an Australian multi-institutional report, two patients who underwent surgery alone with R0 resections remained disease-free at 26 and 37 months (12). While there are no reports of long-term survivors for patients with primary intracranial disease, aggressive surgical management is crucial for optimal disease management. Published case reports describe aggressive local failure as a cause of death. As with many sarcomas, adjuvant radiotherapy often plays a crucial perioperative role. For intracranial CRS, radiotherapy is generally incorporated into the adjuvant treatment plan following surgical resection, though evidence supporting its efficacy in this location is confined to individual case reports, given the rarity of this tumor (13). Moreover, there are no established dose or fractionation regimens for adjuvant or definitive therapy. After definitive surgical management, adjuvant radiotherapy under the high-grade glioma paradigm is often delivered, although hypofractionated courses may be employed as well. Whole-brain radiotherapy is not typically offered for isolated areas of disease as the toxicities are significant and dose escalation beyond 30 Gy is often required for a therapeutic response. As seen in our patient, adjuvant conventional radiotherapy provided durable local control. With her subsequent cranial pattern of spread, hypofractionated and stereotactic techniques were employed with mixed results. As with most sarcomas, the role of systemic therapy tends to be limited. Systemic therapy for CRS remains a significant clinical challenge. Despite the morphologic similarities to Ewing sarcoma, CRS demonstrates inferior chemosensitivity (12). Ifosfamide, an alkylating agent, is included in National Comprehensive Cancer Network (NCCN)-recommended regimens for soft tissue sarcomas and may have some utility (13, 14). However, systemic responses in CRS are typically short-lived, with early relapse or progression common even among patients receiving multi-agent chemotherapy. Another agent that may be considered is pazopanib, a multitargeted tyrosine kinase inhibitor approved for advanced soft tissue sarcomas following prior chemotherapy based on the PALETTE trial (15). While no CRS-specific data exist for pazopanib, it is listed as a preferred subsequent-line option for general soft tissue sarcomas in the NCCN guidelines and has been used in individual CRS cases (14). There have been some reports of immune checkpoint blockade for treating these sarcomas, but none for intracranial disease, given its rarity (16). Preclinical studies of novel therapeutic pathways for treating tumor entities such as this are ongoing (17–19).

In conclusion, primary intracranial CIC-rearranged sarcomas represent ultra-rare and highly lethal malignancies for which no standardized treatment exists. Here we report a case of this malignancy with disparate intracranial spread. The limitations of our report include a single-patient experience, limited evidence regarding systemic therapy, and the absence of prospective treatment data for this rare entity. Maximal safe surgical resection remains the most critical therapeutic intervention, followed by aggressive radiotherapy. The poor prognosis of these patients underscores the need for biology-driven therapeutic strategies and enrollment in clinical trials. International collaboration and prospective registries for this extremely rare tumor are essential to advance understanding and treatment of this disease.

## Data Availability

The original contributions presented in the study are included in the article/supplementary material. Further inquiries can be directed to the corresponding author.
